# What Is a Biofilm? Lessons Learned from Interactions with Immune Cells

**DOI:** 10.3390/ijms252111684

**Published:** 2024-10-30

**Authors:** Paweł Krzyżek

**Affiliations:** Department of Microbiology, Faculty of Medicine, Wroclaw Medical University, 50-368 Wroclaw, Poland; pawel.krzyzek@umw.edu.pl

**Keywords:** biofilm, extracellular polymeric substance, matrixome, immune cells, host–pathogen interaction

## Abstract

Biofilms are unique, multicellular life forms that challenge our understanding of the microbial functioning. The last decades of research on biofilms have allowed us to better understand their importance in the context of both health and various pathologies in the human body, although many knowledge gaps hindering their correct comprehension still exist. Biofilms are classically described as mushroom-shaped structures attached to the substrate; however, an increasing body of evidence shows that their morphology in clinical conditions may differ significantly from that classically presented. Although this may result partly from the unique physicochemical conditions within the host, the interaction between microbes and immune cells during development of a biofilm should not be underestimated. The current Opinion confronts the classical view on biofilms with the latest scientific research describing the vitality of interactions with immune cells as a modulator of the biofilm phenotype and behavior in clinical conditions.

## 1. Introduction

The original, long-standing definition of a biofilm was “an aggregate of microbial cells surrounded by a self-produced polymer matrix” [1]. The first research article in which this term was used in the context of clinical microbiology was published in 1985 [2]. It was noticed that *Pseudomonas aeruginosa*, a persistent pathogen of many hospital-associated infections, when growing as small aggregates was characterized by a significantly lower sensitivity to translation-targeting tobramycin than its free-swimming forms. While in the 1980s, the concept of a biofilm was known only to a scarce fraction of scientists, nowadays the formation of biofilms is one of the most intensively researched phenomena in microbiology [3,4]. Recently, it was estimated that a biofilm constitutes the basic form of life on Earth, as 40–80% of all cells exist in this structure [5]. Taking into account that a biofilm is the primary form of life, understanding its biology and functions is of paramount importance. Despite significant progress in this sector, there are still many knowledge gaps hindering its correct comprehension [6]. Biofilms are classically described as mushroom-shaped structures attached to the substrate; however, an increasing body of evidence shows that their morphology in clinical conditions may differ significantly from that classically presented [7]. Although this may result partly from the unique physicochemical conditions within the host, the interaction between microbes and immune cells during development of a biofilm should not be underestimated.

Therefore, the current Opinion confronts the classical view on biofilms with the latest scientific research describing the vitality of interactions with immune cells as a modulator of the biofilm phenotype and behavior in clinical conditions.

## 2. The Complexity of Biofilm Composition

Biofilms are multicellular aggregates, in which microbial cells are located in an extracellular polymeric substance (EPS) [8]. EPS constitutes a highly hydrated mixture of various macromolecules, including polysaccharides, proteins, lipids and nucleic acids, although its quantity and composition are dynamic in time [9]. It is worth noting that the dominant part of biofilm biomass are not microbial cells but EPS [8,9], and therefore, the correct understanding of the structure and composition of EPS is crucial in increasing the effectiveness of anti-biofilm therapies. Based on the original definition, a biofilm consists of “a self-produced polymer matrix” [1]. However, is a biofilm in fact always an autonomous entity composed only of microbial components? Many studies assessing the structure of biofilms exposed to immune cells indicate something completely different.

A growing body of scientific data indicates that microorganisms can hijack host components to build biofilms (Table 1). One of the first studies, dating back to 2005, showed that neutrophils can stimulate biofilm formation of *P. aeruginosa*, which uses F-actin and DNA after the lysis of these immune cells for primary adhesion and biofilm development [10]. Later studies by others confirmed these observations, additionally indicating the importance of electrostatic interactions provided by these components as a key element in performing their biofilm-promoting function [11,12,13,14]. Subsequently, two studies, both published independently in 2020, shed new light on the incorporation of neutrophil DNA into *P. aeruginosa* biofilms [15,16]. It was shown that neutrophil-derived DNA constitutes an external, “dead zone” surrounding a biofilm, thus protecting it against phagocytosis and positively charged antibiotics (e.g., tobramycin). Recognizing the structural importance of DNA in a biofilm of *P. aeruginosa*, the authors of the above publications pointed to the possibility of using DNase as an adjuvant of classical antibiotic therapies against this bacterium. A group that could benefit from the use of DNase are people suffering from cystic fibrosis, a genetic disorder of the respiratory system, as *P. aeruginosa* colonizes nearly 80% of patients, exacerbating their disease symptoms and producing difficult-to-treat infections [17]. Indeed, a meta-analysis of clinical trials assessing the effectiveness of dornase alfa, a human recombinant DNase, indicated that this preparation significantly improves lung functioning in people with cystic fibrosis [18].

An example of another microorganism that uses host components to build a biofilm is *Staphylococcus aureus,* an opportunistic pathogen colonizing human skin and mucosal surfaces. This pathogen has the ability to absorb host-derived fibrin in a coagulase-dependent manner and incorporate it into the biofilm structure [19,20,21]. This fibrous structure of a biofilm limits not only the phagocytic activity, but also the sensitivity to cell wall-targeting vancomycin. Interestingly, in a recent study focusing on the pathogenesis of a cardiovascular biofilm of *S. aureus* [22], it was noticed that the incorporation of fibrin strands into the biofilm structure is very dynamic and begins just 3 h after exposure to human plasma. Taking into account the importance of host-derived fibrin as a scaffold of the *S. aureus* biofilm, Zapotoczna et al. [20] demonstrated the usefulness of fibrinolytic agents in the biofilm dispersion in a central venous catheter rat model. Another approach taking advantage of the ability of *S. aureus* to hijack fibrin to strengthen its biofilm was presented in a recent article by Scull et al. [23]. Applying in vitro experiments and a murine dermal wound model, the authors tested the effectiveness of fibrin-based nanoparticles loaded with vancomycin against the developing *S. aureus* biofilm. They proved that fibrin-based nanoparticles reacted with *S. aureus* coagulase and were incorporated into a biofilm, leading as a result to the release of vancomycin and eradication of the biofilm. Similarly to experiments on *P. aeruginosa*, the above-presented studies vividly demonstrate how detailed knowledge of phenomena accompanying microbes in the production of a biofilm within the host can constitute the basis for the design of new, targeted anti-biofilm therapies.

When considering the biofilm structure, it is also worth paying attention to the recently proposed concept of changing the five-stage model of biofilm formation (1. reversible adhesion, 2. irreversible adhesion, 3. microcolony development, 4. macrocolony development, and 5. dispersion) to a three-stage model of its maturing (1. aggregation and adhesion, 2. growth and accumulation, and 3. disaggregation and detachment) [7]. This new concept has foundations in the growing body of scientific data indicating the existence of an alternative, non-adherent form of biofilm, often referred to as a “free-floating biofilm”. This type of structure usually takes the form of microscopic aggregates of 10–20 µm in size, being suspended freely in the host’s body fluids. It is worth mentioning that microbial cells building these free-floating biofilms have all the typical features of adhered biofilms, such as the increased tolerance to antimicrobial substances [24]. So far, the existence of free-floating biofilms has been observed in patients with cystic fibrosis [25,26,27] and joint infections [28,29]. Interestingly, it has been observed that host components, including albumin, fibrinogen and/or hyaluronic acid, participate in the establishment of the free-floating biofilms. This may explain why free-floating biofilms are only observed in in vivo studies or advanced in vitro experiments simulating the host microenvironment. It is also worth mentioning that in contrast to many typical, monomicrobial biofilms developed in laboratories, these produced in physiological conditions are most often composed of many species of microorganisms at the same time, translating not only into metabolic synergism, but also increased abilities to survive environmental stress [30,31].

**Figure 1:**
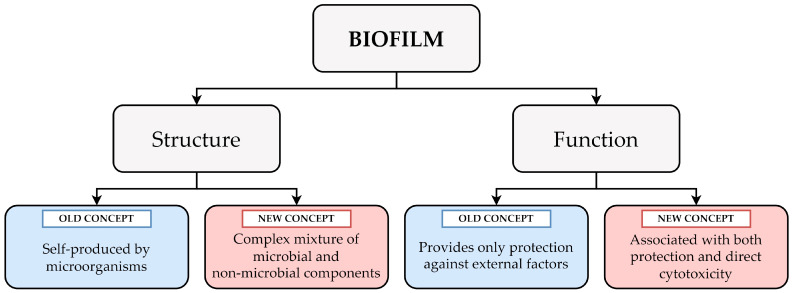
A graph showing the complexity of biofilm and its dual nature at both the structural (a complex mixture of components from microbial and non-microbial origin) and functional (an ability to promote microbial survival and direct cytotoxicity against host cells) levels.

Concluding the examples given above, the author of this Opinion would like to draw attention to the dynamic nature of the biofilm structure (Table 1 and Figure 1). Undoubtedly, in many analyzed laboratory cases where simple culture media are applied, a biofilm can be understood as “an entity autonomously produced by microorganisms”. At the same time, it should be taken into account that in a real-life scenario, a biofilm will constitute a very complex mixture of components of both microbial and non-microbial origin, such as proteins and DNA from lysed immune cells or fibrin coming from the host serum.

## 3. Biofilm—Prey or Predator?

Returning to the concept of the primary function of a biofilm, its ability to limit the contact of EPS-encased microbes with antimicrobial substances and immune cells is classically indicated [8,9,32]. Keeping this in mind, the understanding of a biofilm comes down solely to its capacity to promote microbial survival by restricting direct contact with lethal agents. Is a biofilm actually always only a passive form of protection against external factors, or can it pose a direct threat to the host? Once again, evidence for an alternative role of a biofilm and its involvement in the toxicity toward host cells is provided by studies assessing biofilm–immune cell interactions (Table 2).

The sorptive nature of biofilms determines the accumulation of many different components within EPS [8,9,32]. On the one hand, a high density of EPS slows down diffusion of antibiotics, antibodies or antimicrobial peptides, and protects microorganisms residing within a biofilm. On the other hand, EPS can also store many virulence factors, including toxins and lytic enzymes, which can exert their cytotoxic effect when in close contact with host cells. A perfect example of such substances accumulating specifically in a biofilm are rhamnolipids. These glycolipid surfactants participate in maintaining the appropriate architecture of the *P. aeruginosa* biofilm by affecting the development of water channels and regulating dispersion [33,34]. Interestingly, a biofilm of *P. aeruginosa* senses the presence of leukocytes and, in response to this, increases production of rhamnolipids, leading to disruption of leukocytes by a detergent-like action [35]. This phenomenon was confirmed independently in both in vitro and in vivo studies [11,14,36,37], where it was termed as a “biofilm shield” or “launch a shield” mechanism. A similar process was also observed for *S. aureus*, where a dual nature of its α-hemolysin (structure for a biofilm and cytotoxic against eukaryotic cells) was proved [38,39,40]. It is worth noting that the lytic activity of a biofilm toward immune cells is accompanied by the release of various important macromolecules (e.g., F-actin or DNA) and their incorporation into EPS as described in the previous paragraph. This shows that the direct toxicity against host cells and promotion of survival provided by a biofilm may be much more closely correlated than was initially thought.

The toxicity of a biofilm towards immune cells, however, does not have to be limited to the passive accumulation of toxins in EPS. A groundbreaking discovery was recently made by Vidakovic et al. [41], who focused on the interaction between immune cells and *Vibrio cholerae*, an etiological agent of cholera and one of the model pathogens of complex physiological behaviors. By using a series of detailed in vitro studies and an enteroid-derived human gut model, the team revealed that *V. cholerae* uses biofilm formation as an active process of predation on immune cells. This predatory mechanism occurred on different types of immune cells, including T and B cells, neutrophils, and macrophages, but not monocytes, suggesting that this phenomenon is selective. The initial adhesion of bacteria on the surface of immune cells was associated with the formation of EPS trapping the attacked host cells. This event was accompanied by a hemolysin-dependent lysis of the immune cells and dispersion of bacteria in search of new victims. Interestingly, the composition of EPS necessary to form a biofilm by *V. cholerae* on the surface of immune cells was strikingly different from that classically reported on abiotic surfaces or even epithelial cells.

Considering the above examples, the complexity of biofilm functions might be easier to understand. It should be remembered that these are not limited only to constituting a mechanical barrier against antimicrobial substances or immune cells, but also may be perceived as a phenomenon promoting direct toxicity toward host cells (Table 2 and Figure 1). This cytotoxicity generated by a biofilm can either result from the passive accumulation of lytic factors in EPS or be part of a sophisticated predatory destruction of immune cells.

**Table 2:** Summary of data showing the cytotoxicity of biofilms against host cells.

Author [Reference]	Model Microorganism	Type of Experiment	Type of the Biofilm Cytotoxicity	Interpretation
Jensen et al. [37]	*Pseudomonas aeruginosa*	In vitro + In vivo (murine lung model)	Rhamnolipids accumulate in the biofilm EPS and are toxic against neutrophils	Rhamnolipids participate in the lysis of immune cells and lead to the immune evasion
Alhede et al. [35]				
van Gennip et al. [11]		In vivo (murine model with silicone implants)		
van Gennip et al. [36]		In vitro + In vivo (murine lung model and murine model with silicone implants)		
Watters et al. [14]		In vivo (murine chronic wound model)		Rhamnolipids participate in the lysis of immune cells and allow for stimulation of biofilm formation and antibiotic resistance
Caiazza et al. [38]	*Staphylococcus aureus*	In vitro	α-hemolysin stabilizes biofilm structure	α-hemolysin in a biofilm helps to colonize plastic surfaces
Anderson et al. [39]		In vitro + Ex vivo (porcine vaginal explants)	α-hemolysin stabilizes biofilm structure and is toxic against vaginal mucosal tissue	α-hemolysin in a biofilm helps to colonize vaginal mucosal tissue
Ong et al. [40]		In vitro	α-hemolysin accumulates in a biofilm and is toxic against cancerous human skin cells	α-hemolysin can be used as a good alternative candidate for cancerous human skin treatment
Vidakovic et al. [41]	*Vibrio cholerae*	In vitro, including an enteroid-derived human gut model	Direct attack and biofilm formation on the surface of immune cells	Biofilm formation as a predatory mechanism on immune cells

## 4. Conclusions

Biofilms are unique, multicellular life forms that challenge our understanding of the microbial functioning. The last decades of research on biofilms have allowed us to better understand their importance in the context of both health and various pathologies in the human body. The development of knowledge regarding biofilms will undoubtedly lead to alterations in the originally established terms and dogmas associated with this phenomenon. Considering the increasing body of data on biofilm–immune cell interactions, the author of this Opinion would like to sensitize the scientific community to the need for better adaption of definitions connected with a biofilm. To achieve this task, future research aiming at understanding the function and structure of biofilms should strive to transform in vitro studies in simple culture media into either advanced in vitro and ex vivo research mimicking the host microenvironment or in vivo studies analyzing in detail the interactions between biofilms and the immune system.

## Figures and Tables

**Table 1:** Summary of data showing the structural importance of host-derived components in biofilm formation.

Author [Reference]	Model Microorganism	Type of Experiment	Observations	Interpretation
Walker et al. [10]	*Pseudomonas aeruginosa*	In vitro	F-actin and DNA from lysed neutrophils stimulate primary adhesion of bacteria	Necrotic neutrophils can serve as a biological matrix to facilitate biofilm formation
Parks et al. [12]				
Caceres et al. [13]			F-actin and DNA from lysed neutrophils stimulate autoaggregation of bacteria	Necrotic neutrophils promote bacterial autoaggregation and antibiotic resistance
van Gennip et al. [11]		In vivo (murine model with silicone implants)	DNA from lysed neutrophils stimulates biofilm formation	Rhamnolipids participate in the lysis of immune cells and allow for stimulation of biofilm formation
Watters et al. [14]		In vivo (murine chronic wound model)		Necrotic neutrophils promote biofilm formation and antibiotic resistance
Alhede et al. [15]		In vivo (murine implant model and lung tissues from cystic fibrosis patients)	DNA from lysed neutrophils creates “dead zone” around the biofilm	“Dead zone” from DNA of lysed neutrophils may protect biofilms from harsh conditions
Thanabalasuriar et al. [16]		In vivo (murine keratitis model)		“Dead zone” from DNA of lysed neutrophils protects against antibiotics and neutrophil killing
Kwiecinski et al. [19]	*Staphylococcus aureus*	In vitro + In vivo (murine catheter model)	Host-derived fibrin is used to stimulate biofilm formation	Fibrin-based biofilms are better protected against antibiotics and neutrophil killing
Zapotoczna et al. [20]				Fibrin-based biofilms are better protected against antibiotics
Loof et al. [21]		In vitro + In vivo (murine skin infection model)	Host-derived fibrin is used to stimulate autoaggregation and biofilm formation	Fibrin-based biofilms are better protected against activity of immune cells
Oukrich et al. [22]		In vitro	Host-derived fibrin is used to stimulate biofilm formation	Fibrin strands begin to be incorporated into a biofilm after just 3 h of exposure to human plasma
